# Quality of family planning services for women of reproductive age in Lao PDR

**DOI:** 10.1080/16549716.2020.1788261

**Published:** 2020-08-03

**Authors:** Souksamone Thongmixay, Tess Schoneveld, Viengnakhone Vongxay, Jacqueline E. W. Broerse, Vanphanom Sychareun, Dirk R. Essink

**Affiliations:** a Faculty of Public Health, University of Health Sciences, Vientiane, Lao PDR; b Athena Institute, Faculty of Science, Vrije Universiteit van Amsterdam, Amsterdam, Netherlands

**Keywords:** LEARN: Sexual Reproductive Health, ANC and Nutrition, Contraception, family planning services, unmet need, quality of care, quick investigation of quality

## Abstract

**Background:**

In Lao PDR, 15% of the married women want to postpone or prevent having a child, yet most are not using contraceptives to achieve this. Literature shows that usage of contraceptives is strongly dependent on the quality of family planning services. However, little is known about the quality of family planning services in Lao PDR.

**Objective:**

To assess the quality of family planning services provided in public health facilities in Lao PDR.

**Methods:**

Using a cross-sectional study design, public health facilities in three provinces in Lao PDR were assessed on structure, process and outcome measures of quality. Following the Quick Investigation of Quality approach, client exit interviews (n = 393), structured observations (n = 218) and facility audits (n = 17) were conducted.

**Results:**

Facility audits, observations and client exit interviews painted different pictures of the overall quality of family planning services. Taking all together, the quality was rated as moderate to high. Only marginal differences in quality were found between family planning services located in different geographical areas. Notably, only married women with children were using these services. Although contraceptives were provided, little attention was given to the information provided during consultations and to the interpersonal relationship between client and provider.

**Conclusion:**

The results suggest that although improvements are needed to enhance quality of individual consultations, the greatest gain in reducing unwanted pregnancies would be made by ensuring access for all women of reproductive age.

## Background

Family planning can reduce maternal morbidity and mortality [1]. Furthermore, family planning could limit the numbers of high-risk pregnancies, reproductive tract infections and sexually transmitted diseases [2]. Almost 1 in 6 (14.3%) women in Lao PDR who are married or live in a union want to postpone or prevent having a child, yet most are not using contraceptives to achieve this [3]. Because abortion is restricted in Lao PDR, women with unintended pregnancies may resort to unsafe abortions, which can have dangerous consequences, such as uterine perforation, haemorrhage, and infection [4].

There is evidence that next to barriers for contraceptive use, access to family planning services and the quality of family planning services influences perception and consistent use of contraceptives [5–7]. Because of its impact on contraceptive use, the quality of family planning services is a frequently studied topic [8–12]. The UNFPA conducted an assessment of the quality of family planning services (FPS) in the five (of 18) provinces in Lao PDR where UNFPA is working, and found that the quality of service delivery varied, and was affected predominantly by the communication and clinical skills of service providers as well as the supplies of materials [13]. That study included a facility audit and interviews with health providers from village up to national level. However, it did not include observations of consultations nor client exit interviews to understand the quality of care from the point of view of the users [13]. More direct methods of assessing quality in randomly selected provinces could provide new insights into FPS quality across the country.

This study aims to assess the quality of family planning services provided in public health facilities in the three geographical regions of Lao PDR. The results of this study will be relevant for improving family planning policies and interventions in Lao PDR and form a benchmark for monitoring progress of quality of family planning. The results may also be informative for other countries where uptake of FPS is less than optimal.

## Methods

The Lao Ministry of Health provides FPS in the public sector, situated in Mother and Child Health (MCH) departments at central, provincial and district health facilities [13]. We conducted a public health facilities-based descriptive cross-sectional in three provinces using the Quick Investigation of Quality (QIQ) approach, specifically designed for low- and middle-income countries, to assess the quality of family planning services in terms of structure, process and outcome measures.

### Study site

**Figure 1.**
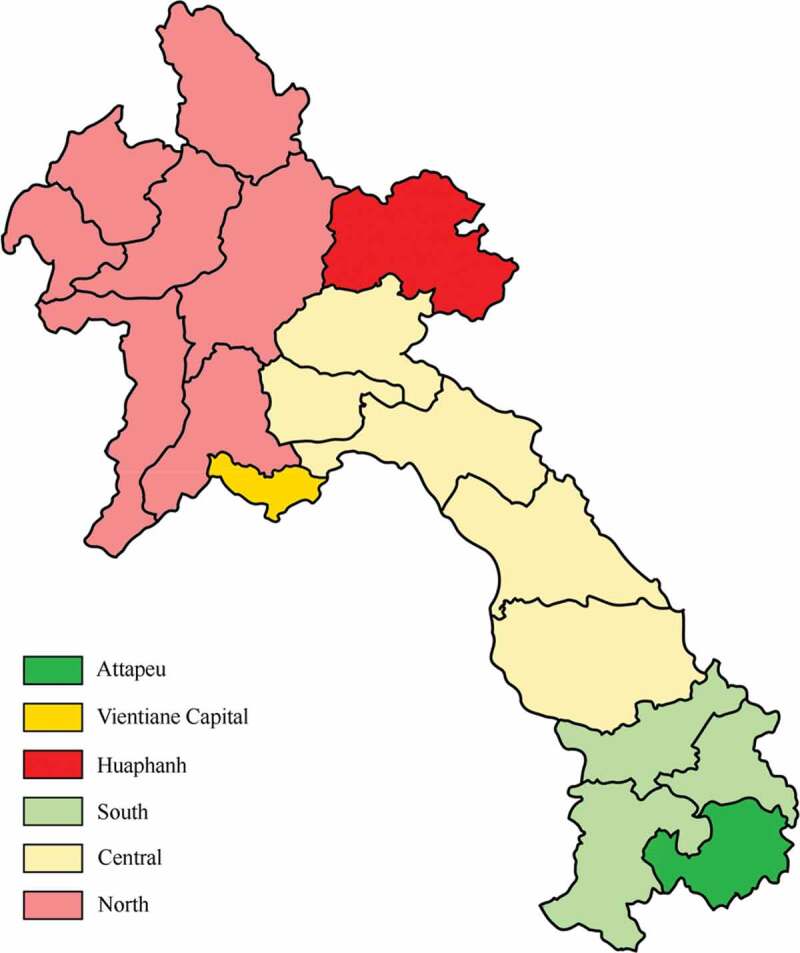
Map of Lao PDR showing the three study provinces (Attapeu, Vientiane Capital, Huaphanh) and the three regions (north, central and south).

There are 18 provinces in Lao PDR: 8 in the North, 4 in the South and 6 in the Central region. A previous assessment with similar objectives was done using the QIQ tool, but only in five provinces that have internationally supported programme interventions for family planning [13]. We therefore selected three provinces from the other 13 provinces where there are only public services, one from each region (Figure 1). The selection was based partly on convenience (Vientiane is both the location of the capital city and in the Central region) and partly by random selection from the remaining provinces in each of the other two regions. Huaphanh was selected in the North and Attapeu in the South; each of these two provinces has only one provincial hospital. In Vientiane, we chose the central-level hospital specialised in mother and child care. The district hospitals were selected purposively by location. In each province, one district hospital was located in a rural area, and the other in an urban area. We selected those which were at similar distance from the provincial hospital, but not too close to international borders. Health centres were selected from among those supervised by the chosen district hospitals. Convenience sampling was used, since some health centres were located in such remote areas that they would have been too time-consuming to reach. When a health centre was under the oversight of an urban district hospital but was located in a rural area, it was still considered urban. See Figure 1 for a map of Laos and the three regions where the study was done.

### Study population

The study population included women of reproductive age (15–45 years) who were the clients of public health services. Data on the number of these women were taken from provincial hospital statistics.

The sample size calculation for exit interviews was based on the estimated number of married reproductive-aged woman in the three provinces accessing the public health facilities from February to June 2017, then assuming 95% standard deviations, 5% acceptable tolerances, and that 37% of the women used a modern contraceptive method provided by the public health facilities [1] and 10% for absolute precision, resulting in a required sample size of 393 FP service clients.

Women were approached at both FP service facilities and in the community. In the service facilities, we invited clients who came for FPS (218) or for MCH (30) services (child vaccination and gynaecological and/or PNC check-up) to participate in the interviews. None of the women refused to be interviewed. Observation was performed during the service provided to each of the 218 clients who came for FPS at a selected facility during the day that the research team was at that facility. The number of women who would attend those services was not known ahead of time. Before the start of observation, the providers agreed to the presence of the observer, who made every effort to stay in the background and did not have contact with either the client or the provider. The observer wore the medical student uniform and remained unobtrusively in the room to observe until the client–provider interaction was finished. In the community, the research team worked with village health volunteers to identify reproductive age women who had ever used the FPS; those who had received FP services in the past 6 months at the selected facility were invited to participate in the study. None of the women who were invited declined to be interviewed. All of the women were given a small gift after the interview or observation session to express our thanks for their willingness to give their time and information.

### Study tools

The Quick Investigation of Quality (QIQ) is a facility-based survey intended to provide a comprehensive picture of family planning services (FPS) in low- and middle-income countries, developed as the latest tool for FPS evaluation for ‘MEASURE Evaluation’ in 2013 by USAID [14–17]. It uses three different measurement tools: facility audits, structured observations and exit interviews with clients [14]. Structural, process and outcome measures of quality were operationalised into five concepts: access, choice of method, information given, technical competence, and interpersonal relationships.

Facility audits were used to obtain information about structural components of quality (i.e. to gain insight into the readiness of facilities to deliver family planning services). Observations were used to gain insight in process outcomes. Family planning services such as consultations and clinical procedures were observed to learn more about how family planning services were delivered to clients. The third tool, client exit interviews, was used to gain insight into structure, process and outcome measures from the clients’ perspective. The tools are provided as supplementary material. The tools were adapted slightly for the local context. All the tools were translated through back and forth translation. Several questions were added to the client exit interviews to gain more insight into the study population and how they perceived services (i.e. client satisfaction). The questions added to the original design are highlighted in grey in the supplementary files. The questionnaire was piloted in a hospital not included in the study (n = 30). The pre-test was done to make sure that clients understood the added questions correctly and that the questions were formulated clearly in the Lao language. Table 1 provides an overview and shows which indicator was measured by which measurement instrument (i.e. facility audit, observation or client exit interview) (Table 1).

**Table 1.** Overview of the quality indicators measured in a QIQ (quick investigation of quality) survey based on 393 family planning service consultations, Lao PDR.

		Facility audit	Observation	Client exit interview
Access				
26	Physical access	x		x
27	Economical access			x
Choice of methods				
4	Provider discusses with client which method she would prefer			x
16	Client receives het choice of method			x
18	Facility has all methods available; no stock-outs	x		
Information given				
5	Provider mentions HIV/AIDS		x	x
6	Provider discussed dual method use		x	x
8	Provider tailors key information to the particular needs of specific client			x
9	Provider gives accurate information on the method accepted		x	x
10	Provider gives instructions on when to return		x	x
Technical competence				
11	Provider follows infection control procedures outlined in guidelines		x	
12	Provider identifies contraindications consistent with guidelines		x	
13	Provider performs clinical procedures according to guidelines		x	
19	Facility has basic items needed for delivery of methods available	x		
22	Facility has received a supervisory visit in past six months	x		
23	Facility has adequate storage of contraceptives and medicines	x		
24	Facility has state-of-the-art clinical guidelines	x		
Interpersonal relationship				
1	Provider demonstrates good counselling skills		x	x
2	Provider assures client of confidentiality		x	
3	Provider asks client about reproductive intentions		x	x
7	Provider treats client with respect/courtesy			x
14	Staff treats client with dignity and respect			x
15	Client participates actively in discussion and selection of method		x	x
17	Client believes the provider will keep her information confidential			x
20	Facility offers privacy for pelvic exam/IUD insertion	x	x	
25	Waiting time acceptable	x		x
Additional				
21	Facility has mechanisms to make programmatic changes based on client feedback	x		

### Data collection

Data were collected between April and July 2017. An all-female study team was trained to conduct the survey according to the QIQ training manual. The reason for an all-female team was to ensure cultural acceptance for the interviews with female subjects. The QIQ data collection tools were reviewed prior to the training, and minimal adaptations were made to fit local context. The physician team leader trained another medical doctor to conduct the observations and medical students to conduct exit interviews. The same team collected the data in all three provinces. The team leader took responsibility for the facility audit while the medical students did exit interviews at a location with sufficient privacy. The doctor obtained verbal consent from the FP provider(s) to observe client visits without interfering.

### Data analysis

Both descriptive and comparative analyses were performed using IBM SPSS Statistics 24. Descriptive analysis was used to gain insight into the overall quality of FP services and to determine how well these services performed on quality indicators. All 28 quality indicators were examined and given a quality indication (high, moderate or low). Scores below 33% were considered low, scores between 34%-66% were considered moderate, and scores of 67% and above were considered high, following the approach used by Simbar, Ahmadi, Ahmadi & Majd [18].

## Results

### Background information

Altogether, 17 facility audits, 218 structured observations and 393 client exit interviews were conducted. Nearly two thirds (63%) of clients were recruited at the FP service facilities. Looking at the characteristics of clients using these FP services, key findings were: the great majority of clients were between 25 and 36 years, with a mean age of 30.9 years. Just over half of the participants (51.4%) had elementary education level or lower. Nearly all the clients were married (96.7%) and 99.2% already had children (Table 2).

**Table 2.** Characteristics of 393 clients attending 17 public family planning services in three provinces in Lao PDR.

	Results n (%)
**Age** < 18 years	4 (1.0)
18–24	72 (18.3)
25–35	219 (55.7)
36–50	98 (24.9)
**Religious belief**	
Buddhism	275 (70.3)
Animism	110 (28.1)
Other	8 (1.6)
**Education**	
No schooling	81 (20.6)
Elementary school	121 (30.8)
Junior high school	96 (24.4)
Senior high school	43 (10.9)
College	34 (8.7)
University	18 (4.6)
**Marital status**	
Married	377 (96.7)
Cohabiting	8 (2.1)
Single, never married	5 (1.3)
**Has children**	
Yes	390 (99.2)
No	3 (0.8)
**Number of living children, mean (range)**	2.8 children (1–7)
**Household income per month, median (IQR)**	1,800,000 kip (100,000–20,000,000)
< 850,000 kip	116 (29.8)
850,001–4,250,000 kip	229 (58.9)
4,250,001–8,500,000 kip	33 (8.5)
> 8,500,000 kip	11 (2.8)

(1 US$ is 850 kip)

### Facility quality according to key features in QIQ

#### Access

Only two of the 17 facilities had a guidance sign on display, announcing the availability of FP services. Less than a quarter of the clients paid for the method received (21.4%). Furthermore, Chi-square testing revealed that clients who received a long-term contraceptive were more likely to pay for the services and method received. Clients who obtained an IUD were more likely to pay for services (p = 0.001) and the devices/medicines (p < 0.001), than clients who received another method. Clients who received an Implanon® were also more likely to pay for the materials (p < 0.001).

#### Choice of methods

**Figure 2.**
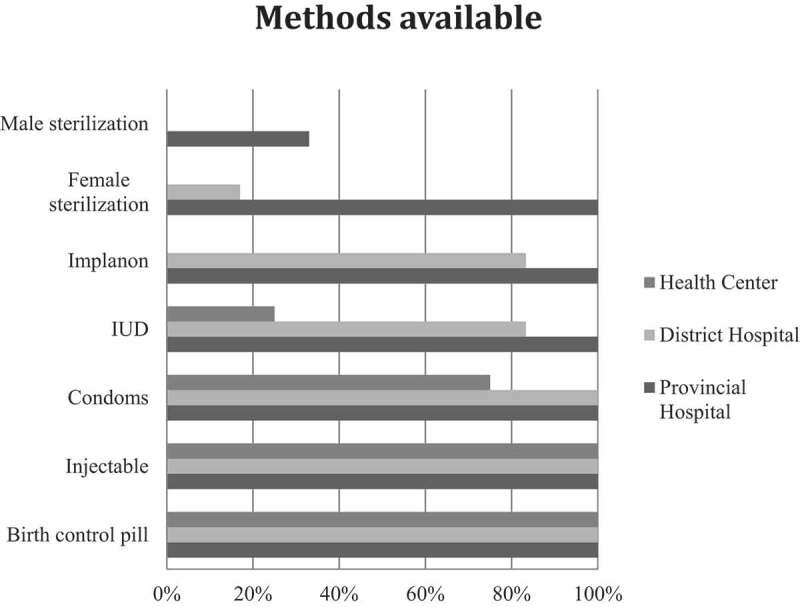
Availability of different FP methods at different levels of health facilities.

Birth control pills and injectables were usually available in all facilities. Long-term methods such as IUDs, Implanon®, female sterilisation and male sterilisation could only be obtained in district hospitals or provincial hospitals, with the exception of two health centres that also provided IUDs. During the facility audit, no stock-outs were observed; however, service providers mentioned that in the 6 months prior to this study, birth control pills, injectables, and condoms were not always available. At the facilities that provided these contraceptives, pills were available 88.2% of the time, injectables 94.1% of the time, and condoms 80% of the time. Of the facilities that experienced stock-outs, 46.7% had a replacement in less than 1 week and 40% in 1 month or less. In total, only 13.3% of the facilities had birth control pills, injectables, condoms, and IUDs available at all times (Figure 2).

#### Information given to clients

**Figure 3.**
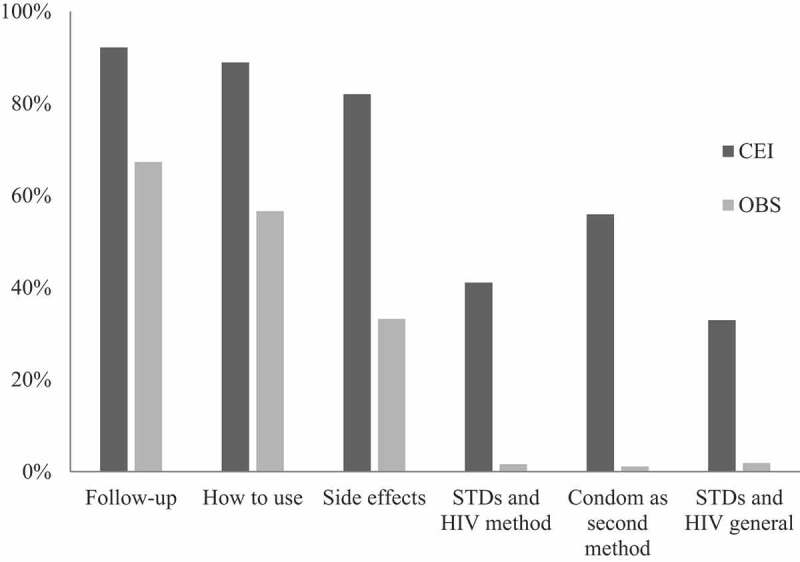
Information discussed during consultations, from client exit interviews (CEI) and observed during consultation (OBS).

With regard to the information given during consultations, the results of client exit interviews contrasted with those from observation. In exit interviews, clients said that essential information about the contraceptive provided and its use was given during the consultation. However, the observations painted a different picture: all topics were discussed to a much lesser extent than clients thought; especially information related to STDs and HIV/AIDS was rarely discussed (Figure 3).

#### Technical competence

In 12 facilities, providers indicated that they had guidelines and protocols on how to deliver FPS to ensure quality service delivery. However, only two facilities could show the protocol when asked and both were based in Vientiane. The guidelines of the QIQ recommend checking blood pressure, checking for pregnancy, asking about smoking, and asking about breastfeeding before providing birth control pills. Blood pressure was only checked in 23.9% of the clients who received birth control pills, and pregnancy in 21.8% of them; only 1.2% was asked about their smoking behaviour, and 12.9% about breastfeeding.

#### Interpersonal relationships

A large proportion of the clients believed that others could overhear their consultation (46.9%) or did not know whether someone could overhear the consultation (15.1%). Furthermore, although during 94.9% of the consultations the service provider said that confidentiality was ensured, only 59.5% of the clients felt that the information they provided would be kept confidential. Of the remaining clients, 15.8% believed that the information they provided would not be kept confidential, and 24.7% did not know whether that would be the case, indicating a lack of trust in the confidentiality capacity of service providers. Little privacy was offered during family planning consults. Observation revealed that only 64.4% of the clients were seen in private settings, for example, the operating room. During pelvic exams and IUD insertions, even fewer clients were offered privacy, only 46.7%; privacy was achieved by doing the examinations in a separate room with no visibility from outside (26.7%), behind a curtain (6.7%), or in the general operating room (13.3%).

### Difference in terms of quality

Analysis revealed that family planning services provided in rural and urban areas differed in four of the 28 quality indicators. Three indicators related to the information provided during a consultation and one to the interpersonal relationship between client and provider, as shown in Table 3. It is notable that clients who received services in a facility located in an urban area were more often 1) advised on how to reach shared-decision making, 2) informed on how to use the provided the contraceptive method, the use of the materials and devices, 3) told what side-effects could be expected, and 4) told when replacement or resupply of the contraceptive would be needed. Furthermore, these clients were more often seen in private compared to clients at a facility located in a rural area.

**Table 3.** Significant differences in quality indicators between rural and urban family planning services, observed on 393 client consultations, Lao PDR.

	Client exit interviews			Observations		
Quality indicators	Rural	Urban	p	Rural	Urban	p
Provider mentions HIV/AIDS						
Advised on shared decision-making	36.9%	64.4%	0.008	-	-	-
Provider gives accurate information on the method						
How to use the provided method	-	-	-	32.7%	65.8%	0.011
Side-effects of the provided method	-	-	-	16.4%	39.6%	< 0.001
Provider gives instructions on when to return						
When re-supply or replacement is needed	41.1%	75.5%	0.005	-	-	-
Provider displays good counselling skills						
Sees client in private	-	-	-	42.9%	71.7%	0.001

## Discussion

The study has provided an insight into the quality of public FPS in Lao PDR in both rural and urban settings using the QIQ tool. The results show that nearly all FPS clients were married women, aged over 18 years and with at least one child. These findings suggest that family planning services in Lao PDR are not accessed by youth, unmarried women, or women without children. Other studies provide evidence that confirms this finding [19–21] which may arise for multiple reasons. Firstly, cultural aspects are likely to contribute, as using family planning services is associated with being sexually active. Sexual activity is not uncommon among youth [19,21,22], yet, in Lao culture, it is not acceptable to have sex before marriage. Therefore, youth and unmarried women might be reluctant to use these public family planning services. A second reason could be that providers impose restrictions to the access of adolescents to family planning services. A third reason could be that adolescents in Lao PDR could experience other barriers related to contraceptive use; a previous study found that adolescents reported barriers such as limited numbers of youth-friendly facilities, geographical difficulties, poverty and their own lack of SRH knowledge and information on where to access services [23]. For example, adolescents were more affected by the distance to a facility than were older women. Fourthly, the referral system in health facilities could contribute to the fact that the services are mostly used by women with children. In Lao PDR, women are referred to family planning services from antenatal care services after childbirth. Possibly, these clients were unaware that family planning services could be obtained before their first child was born. Moreover, family planning services were often located in the Mother and Child Department of the health facility. It is possible that women without children do not attend services in that department. All in all, current family planning services are not providing sexual education or contraceptives in a way appropriate for youth and unmarried women. The study thus provides answers to questions under Topic 5 of the *Health Research Agenda for Lao PDR* [24].

We noted that the different assessment tools used in this study led to different conclusions regarding the quality of family planning services. Facility audits, observations and client exit interviews showed differences in quality results. This could be expected since the tools measure different quality indicators. However, it is important to note that observations and client exit interviews resulted in completely opposing pictures about a few of the same quality indicators. This is especially important because it has been suggested that structured observations and client exit interviews of the QIQ could be used interchangeably [25]. Although they advise using both tools for a comprehensive assessment of quality, Bessinger & Bertrand considered that they would generate comparable results [26]. That was not the case in our study. Differences were noted between the results of structured observations and client exit interviews, especially in relation to what was discussed during consults. For example, from exit interviews, most clients reported that they received specific advice from providers based on the FPS guideline, while observation revealed that providers rarely gave such advice to clients during the consultations. Research should be conducted to determine the comprehensive validation of multi-tool consistency.

A known limitation of client exit interviews is courtesy bias, whereby clients are inclined to give socially desirable answers and thereby provide an overly positive image of the situation [5,25]. This could be even more valid for women in Lao PDR, who are culturally less prone to speak their mind. However, other studies have suggested that exit surveys do not provide an oversaturated image and that the concept of courtesy bias is used as a way to explain discrepancies between different methods [27,28]. Moreover, it could be that clients misunderstood the questions. A language barrier could play a role because the interviewers spoke only Lao, the official national language while Lao PDR has many ethnic minorities, many with their own language; not all of them are able to speak the Lao language [29].

Two main areas for the improvement of the quality of family planning services were identified. Improvements are needed with regard to the information provided during family planning consultations and to interpersonal communication. Firstly, based on the observations of provider–client interactions, very little FP Information Education Communication (IEC) materials information was provided. Information is necessary for clients to make an informed decision about what contraceptive method meets their needs. Twenty years ago, no consensus existed about how much and what kind of information is needed to facilitate a contraceptive choice [30]. More recently, Centers for Disease Control and Prevention recommended ‘All women should be counseled about the full range and effectiveness of contraceptive options for which they are medically eligible so that they can identify the optimal method’ [31]. It seems evident that basic information about how to use the contraceptive, what to do in case of a problem, and side effects of the contraceptive should be discussed during consults, especially since one of the largest barriers to contraceptive use is health concerns related to the use of contraceptives [32]. Secondly, observation results revealed that STDs and HIV/AIDS were rarely discussed. Dehne, Snow & O’Reilly [33] concluded that including STD risk education in existing family planning programmes has a positive effect on client satisfaction and clients’ acceptance of contraceptive methods. However, the studies on which these findings were based were mostly conducted in African countries, where HIV/AIDS forms a more significant problem than in Lao PDR [34]. Nonetheless, it could be that clients in Lao PDR would welcome STD risk prevention education in existing family planning services, especially considering that gonorrhoea and chlamydia infection rates are high [35]. Thirdly, it is likely that different clients have different information needs, regarding the amount and the type of information. As many as one third of the clients felt that the information provided during the consultation was too little, whereas others felt that the amount was about right or even too much. Clients visiting a particular health facility for the first time may have different information needs than returning clients. Our results suggested that in fact three topics are discussed less with clients who visited the facility for the first time: what to do in case of a problem; protection of each contraception method against STDs and HIV/AIDS; and general information on STDs and HIV/AIDS. However, these differences were not found to be statistically significant.

The second area needing improvement is the interpersonal relationships between service providers and clients. The results indicated that a substantial number of clients felt that the information they shared during consultations would not be kept confidential, thought that others could overhear their consultation and noted that they were not seen in private. This aspect of family planning services needs improvement because many studies have demonstrated that the maintenance of privacy and assurance of confidentiality are positively associated with client satisfaction and quality of care [8,36–38]. Moreover, assuring confidentiality could be particularly important in public health facilities; Keesara, Juma & Harper, showed that clients in Kenya believed private providers offered better privacy [37]. Also, Hutchinson, Do & Agha found that the chance that confidentiality was insured was higher in private facilities in Tanzania, Ghana, and Kenya [38]. Privacy should be ensured; an environment must be created that enables clients to actively participate during consultations and to make informed contraceptive choices. Not only physical environmental changes need to be made to ensure privacy, service providers must become aware of why privacy is so important in family planning services, and what is to be gained by enabling clients to participate. To achieve this, interventions are needed. Studies have demonstrated that training of either clients or service providers greatly contributes to making family planning consultations a two-way communication stream [39–41].

### Strengths and limitations

The study had some limitations. Although using three different measurement instruments allowed us to construct a comprehensive image of the quality of family planning services in Lao PDR, the conclusions cannot be claimed to be representative because we used a mixed sampling strategy that included purpose and convenience. Nonetheless, the study identified valuable starting points on how to further improve the quality of family planning services. Secondly, we planned to include only clients who had recently received services, to ensure good recall, but the limited utilisation of family planning services in some facilities mean that we finally included clients who had received services up to 6 months prior to data collection. This did not, however, result in different responses between those having just made a visit or those who had gone earlier. Moreover, when comparisons were made between exit interviews and observations, only clients who had participated in both observation and exit interviews were included for analysis (i.e. only clients who received services on the day of data collection).

One advantage of this study is that it was related to a more extensive study of sexual and reproductive health and access to services, so that while these data were being collected, other teams were also collecting data on related topics. The other studies included the sexual and reproductive health literacy of youth, the causes of and barriers in addressing teenage pregnancies, and unmet family planning needs. Combining the results of these studies will provide more comprehensive insights into the sexual and reproductive health situation in Lao PDR and a better contextual understanding of the quality of family planning services and what may need to be improved.

### Conclusion

Public health facilities in Lao PDR provide family planning services of moderate to high quality to a select group of clients. Aspects of care that need improvement was identified. First, the amount of information discussed during consultations was insufficient; clients received minimal method-related information, while STDs and HIV/AIDS were almost never discussed. Furthermore, the interpersonal relationship between client and provider was not good enough. A substantial number of clients felt that the information they provided would not be kept confidential by a service provider, they felt that others could overhear them or observe treatment, as many consultations were not held in a private setting. The findings described here suggest interventions to improve the quality of family planning services that require change but should be possible in the context of Lao PDR.

This study shows that there is a vast benefit to research why family planning services in Lao PDR are not utilised by youth, unmarried women, and women without children. The reproductive needs of these groups should be mapped and based on those results; interventions should be designed, executed and evaluated accordingly in order to assure accessible family planning services for all women of reproductive age.

## Paper context

Quality of family planning services in Lao PDR was moderate to high. However, we also observed that only women who are married and have children are utilising these services and that the prevention of STDs/HIV is hardly discussed. There is a need to improve access to family planning and SRH services for adolescents and unmarried women, and to increase information sharing on the prevention of STDs, while maintaining the existing good qualities of the services.
